# Increased risk of postpartum depression in women with lactational mastitis: a cross-sectional study

**DOI:** 10.3389/fpsyt.2023.1229678

**Published:** 2023-09-01

**Authors:** Fuyi Shen, Xianjin Zhou, Fei Guo, Kechen Fan, Yilu Zhou, Jianhua Xia, Zhendong Xu, Zhiqiang Liu

**Affiliations:** ^1^Department of Anesthesiology, Shanghai First Maternity and Infant Hospital, School of Medicine, Tongji University, Shanghai, China; ^2^Key Laboratory of Receptor Research, Shanghai Institute of Materia Medica, Chinese Academy of Sciences, Shanghai, China; ^3^Shanghai Key Laboratory of Maternal Fetal Medicine, Shanghai Institute of Maternal-Fetal Medicine and Gynecologic Oncology, Shanghai First Maternity and Infant Hospital, School of Medicine, Tongji University, Shanghai, China; ^4^Department of Anesthesiology, Shanghai Pudong New Area People’s Hospital, Shanghai, China

**Keywords:** lactational mastitis, depression, postpartum period, breastfeeding, cross-sectional study

## Abstract

**Background:**

A high incidence of lactational mastitis mainly occurs during the first month of breastfeeding. It may cause severe pain, frustration, fatigue, stress, and breastfeeding concerns. However, few studies investigated the effects of lactational mastitis on postpartum depression. This study investigated the potential association between lactational mastitis and postpartum depression.

**Methods:**

We examined the associations of lactational mastitis with postpartum depression in 1,551 Chinese women. Lactational mastitis was diagnosed by breast specialists. The presence of depression symptoms was evaluated by the Edinburgh Postnatal Depression Scale (EPDS) and Patient Health Questionnaire 9 (PHQ9) at 6 weeks after delivery. Multiple linear regression analysis and multivariable log-binomial regression analysis were performed to estimate the association between lactational mastitis and postpartum depression.

**Results:**

Among the 1,551 mothers, 147 (9.5%) experienced lactational mastitis diagnosed by breast specialists during the postpartum period. Compared with women without lactational mastitis, the proportion of women with depression symptoms was significantly higher (38.1% vs. 27.4%, *p* = 0.008), and the risk of postpartum depression increased by 68% (RR = 1.68, 95% CI, 1.18, 2.40) in women who had experienced lactational mastitis. In addition, the risk of self-harm or suicidal ideation increased by 89% (RR = 1.89, 95% CI, 1.08, 3.29) in women who experienced lactational mastitis. In stratified analysis, the associations of lactational mastitis with postpartum depression appeared stronger among women aged ≥35 years, with maternal comorbidities, and who delivered a female neonate.

**Conclusion:**

The study results suggest that lactational mastitis is a risk factor for depression during the postpartum period. The impact of lactational mastitis on maternal mental health requires further attention.

**Clinical trial registration:**

chictr.org.cn, ChiCTR2000041519.

## 1. Introduction

Lactational mastitis is a common postpartum inflammatory condition in breastfeeding women and mainly occurs during the first month of breastfeeding (1), with an incidence rate of 9–20% (2). Any state of obstruction in milk drainage, such as infrequent feeding and pressure on breast tissue, can result in lactational mastitis (3). Lactational mastitis causes breast pain, frustration, fatigue, stress, and breastfeeding concerns that may increase the risk of postpartum depression (4–6). However, whether lactational mastitis augments depression risk during the postpartum period, particularly among high-risk women, remains unclear.

Postpartum depression is common within the first 12 months after childbirth, which includes major and minor depressive episodes (4, 7), and their incidences have been increasing for decades (8, 9). Untreated depression has a devastating effect on maternal and paternal health (4, 10), as well as the early neurodevelopment of infants (11). Therefore, it is vital to identify the potential risk factors for perinatal depression, such as lactational mastitis.

This cross-sectional study investigated the potential association between lactational mastitis and postpartum depression. In addition, we evaluated the subgroups of pregnant women who were more sensitive to lactational mastitis regarding postpartum depression.

## 2. Subjects and methods

### 2.1. Study population

This cross-sectional study was conducted at China’s Shanghai First Maternity and Infant Hospital. The participants were women who delivered in our hospital from January to April 2021 and completed the follow-up 42 days after delivery. We restricted the participants to women with singleton pregnancies who were 18–45 years old and had a good understanding of written and spoken Chinese. A total of 1,551 women were enrolled, including 147 mothers who had experienced lactational mastitis diagnosed by breast specialists during the postpartum period. Lactational mastitis is currently diagnosed by clinical evaluation based on signs of inflammation, pain, tenderness, heat, and engorgement, and the patients may experience flu-like symptoms (5, 12).

This study was approved by the Human Ethics Committee of the Shanghai First Maternity and Infant Hospital (KS20302) and conducted in accordance with the tenets of the Declaration of Helsinki. All participants signed informed consent.

### 2.2. Outcomes

Outcome measures included depression symptoms, which were evaluated using the Edinburgh Postnatal Depression Scale (EPDS) and the Patient Health Questionnaire 9 (PHQ9) at 6 weeks after delivery. The EPDS is a 10-item self-report questionnaire used explicitly for postpartum women (13). The Chinese version has concurrent validity, with a standardized Cronbach’s α of 0.73 (14). The presence of depression symptoms was defined as an EPDS score ≥ 9 (including minor and major depression), with a sensitivity of 85% and specificity of 82% (15). The PHQ9 is a 9-item self-report questionnaire that evaluates depression symptoms; the American College of Obstetricians and Gynecologists (ACOG) recommends administering PHQ9 to women during the perinatal period (4). The Chinese version has satisfactory concurrent validity, with a standardized Cronbach’s α of 0.86 (16). The presence of depression symptoms was defined as a PHQ9 score ≥ 5, with a sensitivity of 75% and specificity of 90% (4). The PHQ9 is widely used as a general screening tool for depression, including somatic symptoms such as sleep quality, which is considered a risk factor for the negative maternal mental disorder during the postpartum period (17), while the EPDS places less emphasis on somatic symptoms of depression. The degree of agreement between the EPDS and PHQ9 is only 0.5 during the postpartum period, with a discordance of 17% (18, 19). It has been suggested that both tools are categorized differently during the postpartum period (20). Therefore, the present study comprehensively evaluated the potential relationship between lactational mastitis and depression during the postpartum period, using both screening tools.

### 2.3. Covariates

We selected covariates based on previous studies and expert knowledge (21, 22). A structured questionnaire was designed to collect the selected covariates, including maternal age (< 35 or ≥ 35 years), education level (junior high school or below, high school, bachelor’s degree or above), employment status during pregnancy (employed/unemployed), prenatal depression (yes/no), smoking during pregnancy (yes/no), alcohol use during pregnancy (yes/no), nulliparous (yes/no), maternal comorbidities (yes/no, including gestational diabetes mellitus, hypertension, and thyroid disease), delivery modality (cesarean or vaginal delivery), and neonatal characteristics such as preterm birth (< 37-week gestational period), neonatal sex (female/male), and breastfeeding within the first 6 weeks (exclusive, complementary, or never).

### 2.4. Statistical analyses

The general characteristics of the participating women with and without lactational mastitis experience during the postpartum period were compared using the chi-square test. The differences in EPDS and PHQ9 summary scores between the lactational mastitis and non-mastitis groups were compared by the non-parametric test. The differences in the proportions of women with EPDS cut-off scores ≥9 or PHQ9 cut-off scores ≥5 were compared using the chi-square test. The chi-square test was also used to compare the differences in the proportions of women with self-harm or suicidal ideation (with self-harm ideation: item 10 > 0, in EPDS; with suicidal ideation: item 9 > 0, in PHQ9) between the two groups.

A multiple linear regression analysis was performed to evaluate the associations of lactational mastitis with EPDS or PHQ9 summary scores. A multivariable log-binomial regression analysis was conducted to evaluate the relative risk (RR) and 95% confidence interval (CI) for the proportions of women with lactational mastitis presenting with depression symptoms or self-harm/ suicidal ideation. These two regression analyses were adjusted for the following potential confounders: maternal age, education level, employment status, prenatal depression, nulliparity, smoking, alcohol use, maternal comorbidities, delivery modality, preterm birth, neonatal sex, and breastfeeding status.

Stratified analyses were performed to detect whether the associations of lactational mastitis with postpartum depression symptoms differed according to maternal age, employment status, nulliparity, maternal comorbidities, delivery modality, neonatal sex, and breastfeeding status. RRs and 95% CIs were estimated for each subgroup. Finally, sensitivity analysis was performed to address the confounding effects of prenatal depression, smoking/alcohol use, and preterm birth by restricting log-binomial regression analysis to women with no history of prenatal depressive disorder, women with no habit of smoking or alcohol use during pregnancy, or women with full-term birth.

All statistical analyses were performed using R statistical software version 3.2.3 (R Project for Statistical Computing, The R Foundation, Vienna, Austria), and a two-sided value of *p* of <0.05 was considered statistically significant.

## 3. Results

### 3.1. Demographic characteristics

A total of 1,551 pregnant women with singleton pregnancies were included in this study. The demographic characteristics are summarized and compared in Table 1. Of the participating women, the majority were aged <35 years (85.7%), nulliparous (72.4%), had a bachelor’s degree or above (88.7%), employed during pregnancy (79.2%), and had exclusive or complementary breastfeeding status (93.5%). Only a small number of participating women smoked (0.7%) or consumed alcohol (2.1%) during pregnancy, had a history of prenatal depression (0.5%), and delivered prematurely (7.0%). Among the 1,551 participants, 9.5% (*N* = 147) experienced lactational mastitis during the postpartum period, and the demographic characteristics of these women were not significantly different from those of women who did not experience lactational mastitis.

**Table 1:** Participant demographic characteristics.

Characteristics	Total (*N* = 1,551)	Lactational mastitis		*p*-value
		No (*N* = 1,404)	Yes (*N* = 147)	
Maternal age				0.39
*<35 years*	1,329 (85.7%)	1,207 (86.0%)	122 (83.0%)	
*≥35 years*	222 (14.3%)	197 (14.0%)	25 (17.0%)	
Education levels				0.19
*Junior high school or below*	60 (3.9%)	56 (4.0%)	4 (2.7%)	
*High school*	115 (7.4%)	109 (7.8%)	6 (4.1%)	
*Bachelor’s degree or above*	1,376 (88.7%)	1,239 (88.2%)	137 (93.2%)	
Employment status during pregnancy				0.52
*Employed*	1,229 (79.2%)	1,116 (79.5%)	113 (76.9%)	
*Unemployed*	322 (20.8%)	288 (20.5%)	34 (23.1%)	
Prenatal depressive disorder	8 (0.5%)	8 (0.6%)	0 (0.0%)	1.00
Smoking during pregnancy	11 (0.7%)	11 (0.8%)	0 (0.0%)	0.61
Alcohol use during pregnancy	32 (2.1%)	26 (1.9%)	6 (4.1%)	0.12
Parity				0.71
*Nulliparous*	1,123 (72.4%)	1,019 (72.6%)	104 (70.7%)	
*Multiparous*	428 (27.6%)	385 (27.4%)	43 (29.3)	
Maternal comorbidities	354 (22.8%)	324 (23.1%)	30 (20.4%)	0.53
Delivery modality				0.67
*Cesarean*	622 (40.1%)	566 (40.3%)	56 (38.1%)	
*Vaginal*	929 (59.9%)	838 (59.7%)	91 (61.9%)	
Preterm births	109 (7.0%)	99 (7.1%)	10 (6.8%)	1.00
Neonatal sex				0.74
*Female*	775 (50.0%)	704 (50.1%)	71 (48.3%)	
*Male*	776 (50.0%)	700 (49.9%)	76 (51.7%)	
Breastfeeding status within 42 days				0.78
*Exclusive*	858 (55.3%)	780 (55.6%)	78 (53.1%)	
*Complementary*	592 (38.2%)	532 (37.9%)	60 (40.8%)	
*Never*	101 (6.5%)	92 (6.5%)	9 (6.1%)	

Data are presented as numbers (%).

### 3.2. Distribution of postpartum depression symptoms

Among the 1,551 participants, the summary score of the EPDS was 5 (interquartile range [IQR] 3–7), and that of the PHQ9 was 2 (IQR 1–4). The proportion of women with depression symptoms was 28.4% (EPDS ≥9 or PHQ9 ≥ 5). Women who had experienced lactational mastitis during the postpartum period reported higher summary scores of the EPDS (5 [IQR 3–7] vs. 6 [IQR 3–8], *p* < 0.01) and PHQ9 (2 [IQR 1–4] vs. 3 [IQR 1–5.5], *p* < 0.01) 6 weeks after delivery than women without experience of lactational mastitis (Table 2). In addition, the proportion of women with depression symptoms was significantly higher in the mastitis group than in the non-mastitis group (38.1% vs. 27.4%, *p* = 0.008), and similar results were also obtained when these two screening tools were considered independently (EPDS ≥9: 23.8% vs. 16.4%, *p* = 0.031; PHQ9 ≥ 5: 33.3% vs. 23.9%, *p* = 0.016) (Table 2). Moreover, women who had experienced lactational mastitis during the postpartum period were found to have higher proportions of self-harm or suicidal ideation (11.6% vs. 6.6%, *p* = 0.026). When self-harm ideation and suicidal ideation were considered separately, self-harm ideation showed marginal significance, while there was no significant statistical difference in suicidal ideation (with self-harm ideation: 8.2% vs. 4.7%, *p* = 0.068; with suicidal ideation: 8.2% vs. 5.2%, *p* = 0.133) (Table 2).

**Table 2:** Median (IQR) of EPDS and PHQ9 summary scores, and numbers (percentages) of women with depression symptoms during the postpartum period.

Presence of depression symptoms^a^	Total (*N* = 1,551)	Lactational mastitis		*p*-value^b^
		No (*N* = 1,404)	Yes (*N* = 147)	
Summary score of EPDS	5 [3–7]	5 [3–7]	6 [3–8]	<0.01
Summary score of PHQ9	2 [1–4]	2 [1–4]	3 [1–5.5]	<0.01
Number of EPDS ≥9 or PHQ9 ≥ 5	441 (28.4%)	385 (27.4%)	56 (38.1%)	0.008
*Number of EPDS ≥ 9*	265 (17.1%)	230 (16.4%)	35 (23.8%)	0.031
*Number of PHQ9 ≥ 5*	385 (24.8%)	336 (23.9%)	49 (33.3%)	0.016
With self-harm or suicidal ideation	110 (7.1%)	93 (6.6%)	17 (11.6%)	0.026
*With self-harm ideation*	78 (5.0%)	66 (4.7%)	12 (8.2%)	0.068
*With suicidal ideation*	85 (5.5%)	73 (5.2%)	12 (8.2%)	0.133

^a^Data are shown as median [IQR] or number (percentage). ^b^The *p*-values were calculated by the t-test or chi-square test. IQR, interquartile range; EPDS, Edinburg Postnatal Depression Scale; PHQ9, Patient Health Questionnaire 9.

### 3.3. Association of lactational mastitis with EPDS and PHQ9 summary scores

In the multiple linear regression analysis, lactational mastitis was significantly associated with higher EPDS and PHQ9 summary scores during the postpartum period. In the adjusted model, compared with women without lactational mastitis experience, the mean EPDS summary score was higher by 1.14 (*β* = 1.14, 95% CI: 0.49–1.80, *p* < 0.01), and the mean PHQ9 summary score was higher by 0.78 (*β* = 0.78, 95% CI: 0.24–1.33, *p* < 0.01) in women who had experienced lactational mastitis after delivery (Table 3).

**Table 3:** Adjusted mean difference (95% CI) in the summary scores of EPDS and PHQ9 associated with women with lactational mastitis experience during the postpartum period.

Presence of depression symptoms	Crude model *β* (95% CI)	*p*-value	Adjusted model *β* (95% CI)	*p*-value
Summary score of EPDS	1.10 (0.43–1.76)	<0.01	1.14 (0.49–1.80)	<0.01
Summary score of PHQ9	0.72 (0.17–1.28)	0.01	0.78 (0.24–1.33)	<0.01

Data are adjusted for maternal age, education level, employment status, prenatal depressive disorder, nulliparity, smoking, drinking, maternal comorbidities, delivery modality, preterm birth, neonatal sex, and breastfeeding status. Adjusted β value of > 0 indicated an increased summary score of the EPDS or PHQ9. Multiple linear regression models were performed to evaluate the β and adjusted β value. CI, confidence interval; EPDS, Edinburg Postnatal Depression Scale; PHQ9, Patient Health Questionnaire 9.

### 3.4. Association of lactational mastitis with the proportion of women with depression symptoms

In the multivariable logistic regression analysis, lactational mastitis was significantly associated with an increased risk of postpartum depression (Table 4). Compared with women without lactational mastitis experience, the risk of postpartum depression (EPDS score ≥ 9 or PHQ9 score ≥ 5) increased by 68% (adjusted RR [RR] = 1.68, 95% CI: 1.18–2.4, *p* < 0.01) in women who had experienced lactational mastitis after delivery (Table 4). The findings were consistent when these two scales were used separately (EPDS score ≥ 9: RR = 1.66, 95% CI: 1.1–2.5, *p* = 0.02; PHQ9 score ≥ 5: RR = 1.65, 95% CI: 1.14–2.38, *p* < 0.01) (Table 4). In addition, lactational mastitis was significantly associated with an increased risk of self-harm or suicidal ideation (RR = 1.89, 95% CI: 1.08–3.29). When self-harm ideation and suicidal ideation were considered separately, the associations of lactational mastitis with self-harm ideation showed marginal significantly (RR = 1.84, 95% CI, 0.96–3.52), while there was no significant association with suicidal ideation (RR = 1.69, 95% CI, 0.89–3.23).

**Table 4:** Adjusted relative risk (95% CI) of the proportion of women with depression symptoms associated with women with lactational mastitis experience during the postpartum period.

Presence of depression symptoms	Crude model RR (95% CI)	*p*-value	Adjusted model RR (95% CI)	*p*-value
Number of EPDS ≥9 or PHQ9 ≥ 5	1.63 (1.14–2.32)	<0.01	1.68 (1.18–2.4)	<0.01
*Number of EPDS ≥ 9*	1.60 (1.06–2.39)	0.02	1.66 (1.1–2.5)	0.02
*Number of PHQ9 ≥ 5*	1.59 (1.1–2.29)	0.01	1.65 (1.14–2.38)	<0.01
With self-harm or suicidal ideation	1.84 (1.07–3.19)	0.03	1.89 (1.08–3.29)	0.03
*With self-harm ideation*	1.80 (0.95–3.42)	0.07	1.84 (0.96–3.52)	0.07
*With suicidal ideation*	1.62 (0.86–3.06)	0.14	1.69 (0.89–3.23)	0.11

Data were adjusted for maternal age, education level, employment status, prenatal depressive disorder, nulliparity, smoking, drinking, maternal comorbidities, delivery modality, preterm birth, neonatal sex, and breastfeeding status. aRR of > 1 indicated an increased proportion of women with depression symptoms. Multivariable logistic regression models were performed to evaluate RR and aRR. CI, confidence interval; RR, relative risk; aRR, adjusted relative risk; EPDS, Edinburg Postnatal Depression Scale; PHQ9, Patient Health Questionnaire 9.

### 3.5. Stratified analysis and sensitivity analysis

We performed a sensitivity analysis to address the impact of confounding effects. By restricting the multiple linear regression analysis to women with prenatal depression, the associations between lactational mastitis and postpartum depression symptoms did not change (Supplementary Table S1). When restricting the analysis to women with smoking/alcohol use habits, the observed associations did not change (Supplementary Table S2). After restricting the analysis to women with preterm births, the associations remained unchanged (Supplementary Table S3).

**Figure 1:**
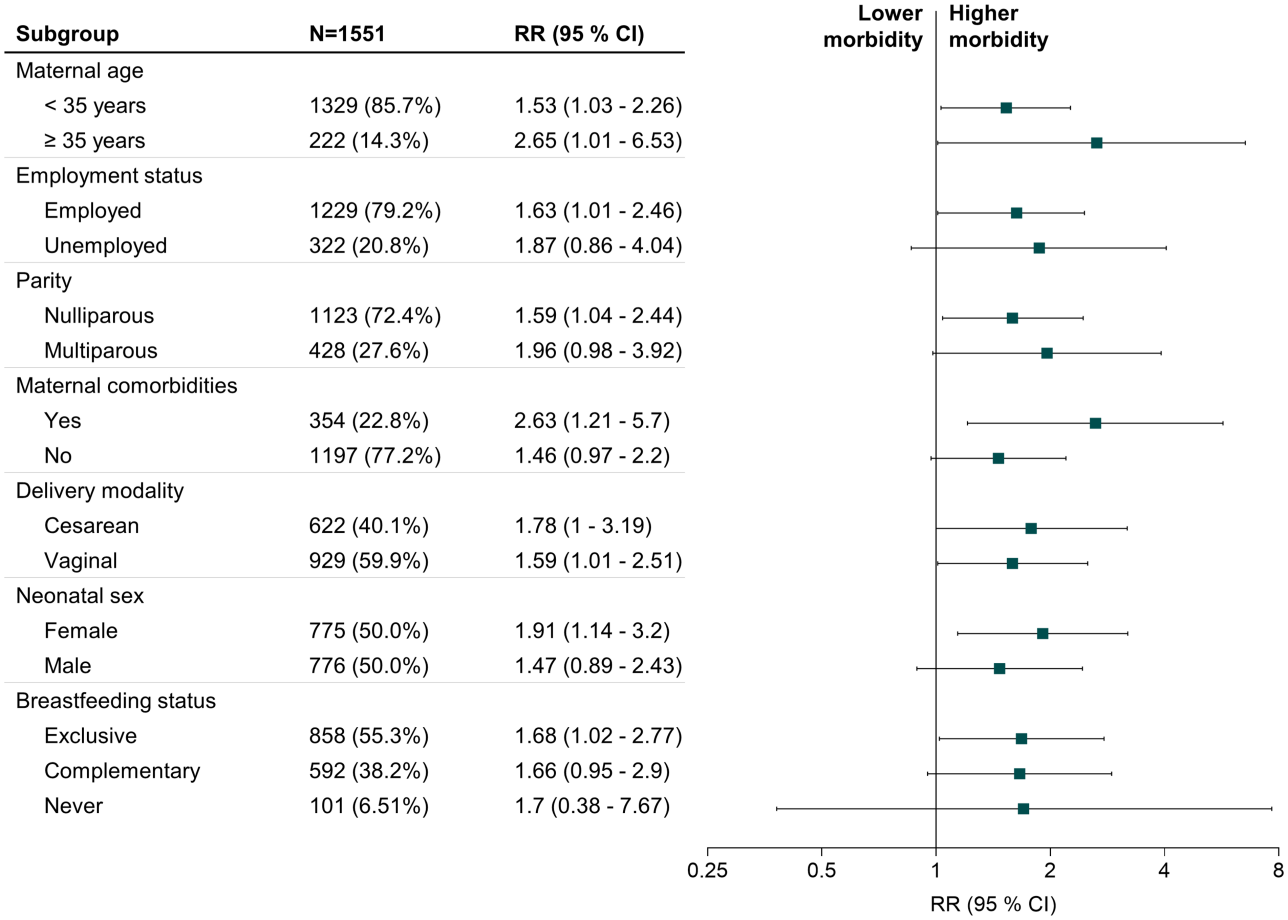
Forest plot of the RR for lactational mastitis associated with postpartum depressive symptoms stratified by influence factors. The stratified analysis indicated that the association of lactational mastitis with postpartum depressive symptoms were stronger in women aged ≥35 years, employed during the perinatal period, with nulliparity, with maternal comorbidities, delivered a female neonate, or exclusively breastfed. Blue squares indicate the RR for influencing factors. Horizontal black lines indicate 95% CI. RR, relative risk; CI, confidence interval.

Further, we performed a stratified analysis by maternal age, employment status, nulliparity, maternal comorbidities, delivery modality, neonatal sex, and breastfeeding status and calculated RRs and 95% CIs for each subgroup (Figure 1). The association between lactational mastitis and postpartum depression symptoms was more robust in participants aged ≥35 years (RR = 2.65, 95% CI: 1.01–6.53, *p* = 0.03), with maternal comorbidities (RR = 2.63, 95% CI: 1.21–5.7, *p* = 0.02), and those who delivered a female neonate (RR = 1.91, 95% CI: 1.14–3.2, *p* = 0.01).

## 4. Discussion

Perinatal depression is a disabling but treatable mental disorder (23). Due to social stigma and prejudice, mothers tend to conceal symptoms, which can result in severe and chronic symptoms (24, 25). Early-stage detection and addressing their symptoms may help reduce severity and chronicity. Therefore, it is crucial to explore potential risk factors for perinatal depression.

In this study, 28.4% of all women enrolled had depression symptoms (EPDS score ≥ 9 or PHQ9 score ≥ 5), of whom 17.1% had an EPDS score ≥ 9 and 24.8% had a PHQ9 score ≥ 5. Our findings are similar to those of previous studies, one of which demonstrated that 17.4% of women had depression symptoms during the postpartum period, detected by EPDS scores ≥9 (26), whereas other studies indicated the proportion varied from 32 to 45.0% detected by PHQ9 scores ≥5 (27, 28). We evaluated the possible association between lactational mastitis and postpartum depression symptoms. We found that lactational mastitis was associated with an increased summary score on the screening scales and an increased prevalence of depression symptoms at 6 weeks after delivery. Moreover, the scores obtained by the EPDS and PHQ9 were consistent, which further enhanced the reliability of the association. After controlling for various covariates, women with lactational mastitis experienced a 68% higher incidence of depression symptoms 6 weeks after delivery. Further, our results indicated that lactational mastitis was associated with an increased risk of self-harm or suicidal ideation. Recent studies suggested that self-harm is a strong predictor of suicide attempts and a more powerful predictor than other suicide risk factors (29). Therefore, our findings provide evidence that lactational mastitis increases the risk of depression and self-harm or suicidal ideation during the postpartum period.

To the best of our knowledge, this was the first study to detect the effect of lactational mastitis on postpartum depression. Therefore, we cannot directly compare our research findings with those of other studies. Lactational mastitis mainly occurs with high morbidity rates of 9–20% in the early stages after delivery. In the present study, 9.5% of participants experienced lactational mastitis during the first 6 weeks, similar to a previous study (2). Severe acute pain experience and perception, rather than the degree of tissue inflammation, are suggested to be associated with an increased risk of postpartum depression (30–32). Frustration is an emotional response to stress. Long-term frustration may lead to other mood disorders, including stress, anxiety, depression, and loss of confidence (33, 34). Stress during the early postpartum period and breastfeeding problems are considered risk factors for postpartum depression by the ACOG Committee’s opinion on perinatal depression (4). Due to a lack of evidence for non-pharmacological prevention and treatment approaches, lactational mastitis is usually treated with antibiotics (5). However, concerns about potential medication exposure to infants through breast milk may further increase maternal anxiety (35, 36).

The stratified analysis showed that the association of lactational mastitis with postpartum depression symptoms appeared to be strong among women with advanced maternal age (≥ 35 years), maternal comorbidities, and those who delivered a female neonate. A cross-sectional study conducted in China reported that advanced maternal age increased the risk of postpartum depression 6 weeks after delivery (37) in accordance with the findings of another study focusing on Chinese women (38). This may be explained by concerns regarding medical risks, difficulty in parenting because they lack friends with children in the same age group, or less family support from much older parents. Two studies involving the Chinese population suggested that delivering a female neonate is associated with postpartum depression (37, 39). However, this finding was not observed in European and American populations. This may be explained by the strong desire for male offspring and the concept of patriarchy, which is common in China and other East Asian countries. Maternal comorbidities during pregnancy are associated with an increased risk of depression symptoms, anxiety, and learning impairments during the postpartum period (22, 40, 41). Women seem to report giving birth as a traumatic event when they have maternal comorbidities (42, 43).

Our findings provide new insights into the positive association between lactational mastitis and the risk of depression symptoms during the postpartum period, indicating that lactational mastitis needs to be taken seriously, particularly in postpartum women. Considering that EPDS and PHQ9 scores may be categorized differently in the postpartum period because of unsatisfactory agreement, we applied both scales to improve the evaluation of the relationship between lactational mastitis and depression during the postpartum period (18–20).

Our study had several limitations. First, it was conducted at a single center, which may limit the generalizability of our findings. Therefore, multicenter studies are needed. Second, this study lacked detailed data on lactational mastitis, including accompanying symptoms, duration, and treatment. Finally, the time point for psychological evaluation was selected only 6 weeks after delivery. Detailed data on lactational mastitis and psychological status at other time points, such as 3 and 6 months after delivery, require further research.

In conclusion, to the best of our knowledge, this study provided the first evidence of lactational mastitis being associated with an increased risk of maternal depression during the postpartum period. Women with advanced maternal age, maternal comorbidities, or delivered a female neonate may be particularly susceptible to the adverse effects of lactational mastitis on mental health and require additional attention. A future multicenter prospective cohort study is necessary to validate our findings.

## Data availability statement

The raw data supporting the conclusions of this article will be made available by the authors, without undue reservation.

## Ethics statement

The studies involving humans were approved by the Human Ethics Committee of the Shanghai First Maternity and Infant Hospital. The studies were conducted in accordance with the local legislation and institutional requirements. The participants provided their written informed consent to participate in this study.

## Author contributions

FS and FG: conceptualization, data curation, methodology, and writing – original draft. XZ: conceptualization, investigation, visualization, and writing – original draft. YZ: conceptualization, investigation, and writing – review and editing. KF: conceptualization, software, formal analysis, and writing – review and editing. JX: conceptualization and writing – review and editing. ZX: conceptualization, formal analysis, supervision, methodology, and writing – review and editing. ZL: conceptualization, project administration, supervision, and writing – review & editing. All authors agree to be accountable for the content of the work.

## Funding

This work was supported by the National Natural Science Foundation (81971418), Science and Technology Commission of Shanghai Municipality (22XD1402400), Pudong New Area Commission of Health and Family Planning (PW2021D-01), Health Science and Technology Project of Health Commission of Pudong New Area (PW2020A-26), and Shanghai Municipal Commission of Health and Family Planning (202140253).

## Conflict of interest

The authors declare that the research was conducted in the absence of any commercial or financial relationships that could be construed as a potential conflict of interest.

## Publisher’s note

All claims expressed in this article are solely those of the authors and do not necessarily represent those of their affiliated organizations, or those of the publisher, the editors and the reviewers. Any product that may be evaluated in this article, or claim that may be made by its manufacturer, is not guaranteed or endorsed by the publisher.

## References

[1] Shalev Ram H Ram S Wiser I Tchernin N Chodick G Cohen Y. Associations between breast implants and postpartum lactational mastitis in breastfeeding women: retrospective study. BJOG. (2022) 129:267–72. doi: 10.1111/1471-0528.16902, PMID: 34486797

[2] Berens PD. Breast pain: engorgement, nipple pain, and mastitis. Clin Obstet Gynecol. (2015) 58:902–14. doi: 10.1097/GRF.0000000000000153 26512442

[3] Schwartz K D'Arcy HJ Gillespie B Bobo J Longeway M Foxman B. Factors associated with weaning in the first 3 months postpartum. J Fam Pract. (2002) 51:439–44. PMID: 12019051

[4] ACOG Committee Opinion No. 757. Screening for perinatal depression. Obstet Gynecol. (2018) 132:e208–12. doi: 10.1097/AOG.0000000000002927 30629567

[5] Barker M Adelson P Peters MDJ Steen M. Probiotics and human lactational mastitis: a scoping review. Women Birth. (2020) 33:e483–91. doi: 10.1016/j.wombi.2020.01.001 32146088

[6] Foxman B D'Arcy H Gillespie B Bobo JK Schwartz K. Lactation mastitis: occurrence and medical management among 946 breastfeeding women in the United States. Am J Epidemiol. (2002) 155:103–14. doi: 10.1093/aje/155.2.103 11790672

[7] O'Hara MW McCabe JE. Postpartum depression: current status and future directions. Annu Rev Clin Psychol. (2013) 9:379–407. doi: 10.1146/annurev-clinpsy-050212-185612, PMID: 23394227

[8] Abel KM Hope H Swift E Parisi R Ashcroft DM Kosidou K. Prevalence of maternal mental illness among children and adolescents in the UK between 2005 and 2017: a national retrospective cohort analysis. Lancet Public Health. (2019) 4:e291–300. doi: 10.1016/S2468-2667(19)30059-3, PMID: 31155222 PMC6557735

[9] Sultan P Ando K Elkhateb R George RB Lim G Carvalho B. Assessment of patient-reported outcome measures for maternal postpartum depression using the consensus-based standards for the selection of health measurement instruments guideline: a systematic review. JAMA Netw Open. (2022) 5:e2214885. doi: 10.1001/jamanetworkopen.2022.14885, PMID: 35749118 PMC9233232

[10] Melrose S. Paternal postpartum depression: how can nurses begin to help? Contemp Nurse. (2010) 34:199–210. doi: 10.5172/conu.2010.34.2.199, PMID: 20509804

[11] Rogers A Obst S Teague SJ Rossen L Spry EA Macdonald JA. Association between maternal perinatal depression and anxiety and child and adolescent development: a meta-analysis. JAMA Pediatr. (2020) 174:1082–92. doi: 10.1001/jamapediatrics.2020.2910, PMID: 32926075 PMC7490743

[12] Cusack L Brennan M. Lactational mastitis and breast abscess – diagnosis and management in general practice. Aust Fam Physician. (2011) 40:976–9. PMID: 22146325

[13] Cox JL Holden JM Sagovsky R. Detection of postnatal depression. Development of the 10-item Edinburgh postnatal depression scale. Br J Psychiatry. (1987) 150:782–6. doi: 10.1192/bjp.150.6.782 3651732

[14] Lee DT Yip SK Chiu HF Leung TY Chan KP Chau IO. Detecting postnatal depression in Chinese women. Br J Psychiatry. (1998) 172:433–7. doi: 10.1192/bjp.172.5.433 9747407

[15] Hewitt C Gilbody S Brealey S Paulden M Palmer S Mann R. Methods to identify postnatal depression in primary care: an integrated evidence synthesis and value of information analysis. Health Technol Assess. (2009) 13:1–230. doi: 10.3310/hta13360, PMID: 19624978

[16] Wang W Bian Q Zhao Y Li X Wang W du J. Reliability and validity of the Chinese version of the patient health questionnaire (PHQ-9) in the general population. Gen Hosp Psychiatry. (2014) 36:539–44. doi: 10.1016/j.genhosppsych.2014.05.021, PMID: 25023953

[17] Okun ML Mancuso RA Hobel CJ Schetter CD Coussons-Read M. Poor sleep quality increases symptoms of depression and anxiety in postpartum women. J Behav Med. (2018) 41:703–10. doi: 10.1007/s10865-018-9950-7, PMID: 30030650 PMC6192841

[18] Flynn HA Sexton M Ratliff S Porter K Zivin K. Comparative performance of the Edinburgh postnatal depression scale and the patient health Questionnaire-9 in pregnant and postpartum women seeking psychiatric services. Psychiatry Res. (2011) 187:130–4. doi: 10.1016/j.psychres.2010.10.022, PMID: 21122923

[19] Yawn BP Pace W Wollan PC Bertram S Kurland M Graham D. Concordance of Edinburgh postnatal depression scale (EPDS) and patient health questionnaire (PHQ-9) to assess increased risk of depression among postpartum women. J Am Board Fam Med. (2009) 22:483–91. doi: 10.3122/jabfm.2009.05.080155, PMID: 19734393

[20] Heck JL. Screening for postpartum depression in American Indian/Alaska native women: a comparison of two instruments. Am Indian Alsk Native Ment Health Res. (2018) 25:74–102. doi: 10.5820/aian.2502.2018.74, PMID: 29889949

[21] Jigeer G Tao W Zhu Q Xu X Zhao Y Kan H. Association of residential noise exposure with maternal anxiety and depression in late pregnancy. Environ Int. (2022) 168:107473. doi: 10.1016/j.envint.2022.107473, PMID: 35994797

[22] der Zee-van V den Berg AI Boere-Boonekamp MM CGM G-O Reijneveld SA. Postpartum depression and anxiety: a community-based study on risk factors before, during and after pregnancy. J Affect Disord. (2021) 286:158–65. doi: 10.1016/j.jad.2021.02.062 33725615

[23] Stewart DE Vigod S. Postpartum depression. N Engl J Med. (2016) 375:2177–86. doi: 10.1056/NEJMcp1607649 27959754

[24] Pinto-Foltz MD Logsdon MC. Stigma towards mental illness: a concept analysis using postpartum depression as an exemplar. Issues Ment Health Nurs. (2008) 29:21–36. doi: 10.1080/01612840701748698, PMID: 18214776

[25] Rahman A Fisher J Bower P Luchters S Tran T Yasamy MT. Interventions for common perinatal mental disorders in women in low- and middle-income countries: a systematic review and meta-analysis. Bull World Health Organ. (2013) 91:593–601I. doi: 10.2471/BLT.12.109819, PMID: 23940407 PMC3738304

[26] Adewuya AO Ola BA Dada AO Fasoto OO. Validation of the Edinburgh postnatal depression scale as a screening tool for depression in late pregnancy among Nigerian women. J Psychosom Obstet Gynaecol. (2006) 27:267–72. doi: 10.1080/01674820600915478, PMID: 17225628

[27] Kroenke K Spitzer RL Williams JB. The PHQ-9: validity of a brief depression severity measure. J Gen Intern Med. (2001) 16:606–13. doi: 10.1046/j.1525-1497.2001.016009606.x, PMID: 11556941 PMC1495268

[28] Sun JW Cao DF Li JH Zhang X Wang Y Bai HY. Profiles and characteristics of clinical subtypes of perinatal depressive symptoms: a latent class analysis. J Adv Nurs. (2019) 75:2753–65. doi: 10.1111/jan.14136 31236991

[29] Halverson TF Patel TA Mann AJD Evans MK Gratz KL Beckham JC. The screen for nonsuicidal self-injury: development and initial validation among veterans with psychiatric disorders. Suicide Life Threat Behav. (2022) 52:615–30. doi: 10.1111/sltb.12847, PMID: 35257418 PMC9378472

[30] Eisenach JC Pan PH Smiley R Lavand'homme P Landau R Houle TT. Severity of acute pain after childbirth, but not type of delivery, predicts persistent pain and postpartum depression. Pain. (2008) 140:87–94. doi: 10.1016/j.pain.2008.07.011, PMID: 18818022 PMC2605246

[31] Lin R Lu Y Luo W Zhang B Liu Z Xu Z. Risk factors for postpartum depression in women undergoing elective cesarean section: a prospective cohort study. Front Med. (2022) 9:1001855. doi: 10.3389/fmed.2022.1001855, PMID: 36250100 PMC9553994

[32] Makeen M Farrell LM LaSorda KR Deng Y Altamirano V Jarvis O. Associations between postpartum pain, mood, and maternal-infant attachment and parenting outcomes. Sci Rep. (2022) 12:17814. doi: 10.1038/s41598-022-21793-1, PMID: 36280697 PMC9592584

[33] Chen X Lu E Stone SL Thu Bui OT Warsett K Diop H. Stressful life events, postpartum depressive symptoms, and partner and social support among pregnant people with disabilities. Womens Health Issues. (2023) 33:167–74. doi: 10.1016/j.whi.2022.10.006, PMID: 36463011

[34] Clancy N Aslam T Cackett P. Depression secondary to vision loss in old age and an effective rapid screening tool for undiagnosed cases. Ann General Psychiatry. (2022) 21:15. doi: 10.1186/s12991-022-00396-0, PMID: 35655227 PMC9160179

[35] Amir LH Griffin L Cullinane M Garland SM. Probiotics and mastitis: evidence-based marketing? Int Breastfeed J. (2016) 11:19. doi: 10.1186/s13006-016-0078-5, PMID: 27446229 PMC4955247

[36] Jahanfar S Ng CJ Teng CL. Antibiotics for mastitis in breastfeeding women. Cochrane Database Syst Rev. (2013) 2013:CD005458. doi: 10.1002/14651858.CD005458.pub3 PMC11297410 23450563

[37] Xiong R Deng A. Incidence and risk factors associated with postpartum depression among women of advanced maternal age from Guangzhou, China. Perspect Psychiatr Care. (2020) 56:316–20. doi: 10.1111/ppc.12430, PMID: 31364779

[38] Jin Q Mori E Sakajo A. Risk factors, cross-cultural stressors and postpartum depression among immigrant Chinese women in Japan. Int J Nurs Pract. (2016) 22:38–47. doi: 10.1111/ijn.12438, PMID: 27184701

[39] Deng AW Xiong RB Jiang TT Luo YP Chen WZ. Prevalence and risk factors of postpartum depression in a population-based sample of women in Tangxia community, Guangzhou. Asian Pac J Trop Med. (2014) 7:244. doi: 10.1016/S1995-7645(14)60030-4 24507649

[40] Koric A Singh B VanDerslice JA Stanford JB Rogers CR Egan DT. Polycystic ovary syndrome and postpartum depression symptoms: a population-based cohort study. Am J Obstet Gynecol. (2021) 224:591.e1–591.e12. doi: 10.1016/j.ajog.2020.12.1215, PMID: 33412131 PMC8285068

[41] Wallace K Bowles T Griffin A Robinson R Solis L Railey T. Evidence of anxiety, depression and learning impairments following prenatal hypertension. Behav Sci (Basel). (2022) 12:53. doi: 10.3390/bs12020053, PMID: 35200304 PMC8869594

[42] Roberts L Henry A Harvey SB Homer CSE Davis GK. Depression, anxiety and posttraumatic stress disorder six months following preeclampsia and normotensive pregnancy: a P4 study. BMC Pregnancy Childbirth. (2022) 22:108. doi: 10.1186/s12884-022-04439-y, PMID: 35130869 PMC8822717

[43] Shuffrey LC Lucchini M Morales S Sania A Hockett C Barrett E. Gestational diabetes mellitus, prenatal maternal depression, and risk for postpartum depression: an environmental influences on child health outcomes (ECHO) study. BMC Pregnancy Childbirth. (2022) 22:758. doi: 10.1186/s12884-022-05049-4, PMID: 36209070 PMC9548153

