# Supplementary material for: Increased risk of postpartum depression in women with lactational mastitis: a cross-sectional study
Source: Front Psychiatry. 2023 Sep 1;14:1229678. doi: 10.3389/fpsyt.2023.1229678 (PMC10506305; doi:10.3389/fpsyt.2023.1229678)
Supplement: Supplementary file 1 [file Table_1.DOCX]

**Table S1** Adjusted relative risk (95% CI) of the proportion of women with depression symptoms associated with women with lactational mastitis experience during the postpartum period among women with no history of prenatal depressive disorder

| **Presence of depression symptoms** | **Crude model**  **RR (95% CI)** | **p-value** | **Adjusted model**  **aRR (95% CI)** | **p-value** |
| --- | --- | --- | --- | --- |
| Number of EPDS ≥ 9 or PHQ9 ≥ 5 | 1.66 (1.16–2.36) | < 0.01 | 1.68 (1.17–2.40) | < 0.01 |
| *Number of EPDS ≥ 9* | 1.62 (1.08–2.43) | 0.02 | 1.66 (1.10–2.50) | 0.02 |
| *Number of PHQ9 ≥ 5* | 1.62 (1.13–2.34) | < 0.01 | 1.64 (1.14–2.38) | < 0.01 |

Note: Data were adjusted for maternal age, education level, employment status, nulliparity, smoking, drinking, maternal comorbidities, delivery modality, preterm birth, neonatal sex, and breastfeeding status. aRR of > 1 indicated an increased proportion of women with depression symptoms. A multiple linear regression analysis was performed to evaluate the RR and aRR.

Abbreviations: CI, confidence interval; RR, relative risk; aRR, adjusted relative risk; EPDS, Edinburg Postnatal Depression Scale; PHQ9, Patient Health Questionnaire 9.

**Table S2** Adjusted relative risk (95% CI) of the proportion of women with depression symptoms associated with women with lactational mastitis experience during the postpartum period among women with no habit of smoking or drinking during pregnancy

| **Presence of depression symptoms** | **Crude model**  **RR (95% CI)** | **p-value** | **Adjusted model**  **aRR (95% CI)** | **p-value** |
| --- | --- | --- | --- | --- |
| Number of EPDS ≥ 9 or PHQ9 ≥ 5 | 1.58 (1.1–2.27) | 0.01 | 1.64 (1.14–2.37) | < 0.01 |
| *Number of EPDS ≥ 9* | 1.61 (1.06–2.43) | 0.03 | 1.67 (1.1–2.55) | 0.02 |
| *Number of PHQ9 ≥ 5* | 1.57 (1.08–2.28) | 0.02 | 1.64 (1.12–2.39) | 0.01 |

Note: Data were adjusted for maternal age, education level, employment status, prenatal depressive disorder, nulliparity, maternal comorbidities, delivery modality, preterm birth, neonatal sex, and breastfeeding status. aRR of > 1 indicated an increased proportion of women with depression symptoms. Multiple linear regression analysis was performed to evaluate RR and aRR.

Abbreviations: CI, confidence interval; RR, relative risk; aRR, adjusted relative risk; EPDS, Edinburg Postnatal Depression Scale; PHQ9, Patient Health Questionnaire 9.

**Table S3** Adjusted relative risk (95% CI) of the proportion of women with depression symptoms associated with women with lactational mastitis experience during the postpartum period among women with full-term births

| **Presence of depression symptoms** | **Crude model**  **RR (95% CI)** | **p-value** | **Adjusted model**  **aRR (95% CI)** | **p-value** |
| --- | --- | --- | --- | --- |
| Number of EPDS ≥ 9 or PHQ9 ≥ 5 | 1.66 (1.15–2.39) | < 0.01 | 1.71 (1.18–2.47) | < 0.01 |
| Number of EPDS ≥ 9 | 1.51 (0.98–2.31) | 0.06 | 1.55 (1.01–2.4) | 0.05 |
| Number of PHQ9 ≥ 5 | 1.64 (1.13–2.4) | 0.01 | 1.7 (1.56–2.48) | < 0.01 |

Note: Data were adjusted for maternal age, education level, employment status, prenatal depressive disorder, nulliparity, smoking, drinking, maternal comorbidities, delivery modality, neonatal sex, and breastfeeding status. aRR of >1 indicated an increased proportion of women with depression symptoms. Multiple linear regression analysis was performed to evaluate RR and aRR.

Abbreviations: CI, confidence interval; RR, relative risk; aRR, adjusted relative risk; EPDS, Edinburg Postnatal Depression Scale; PHQ9, Patient Health Questionnaire 9.
